# Supplementary material for: The ATR Inhibitor VE-821 Enhances the Radiosensitivity and Suppresses DNA Repair Mechanisms of Human Chondrosarcoma Cells
Source: Int J Mol Sci. 2023 Jan 24;24(3):2315. doi: 10.3390/ijms24032315 (PMC9917087; doi:10.3390/ijms24032315)
Supplement: Supplementary file 1 [file ijms-24-02315-s001.zip › ijms-2127178-Supplementary.pdf]

SW-1353 1h	ctrl 0 Gy	Ola 0 Gy	Veli 0 Gy	ctrl 4 Gy	Ola 4 Gy	Veli 4 Gy	ctrl 8 Gy	Ola 8 Gy	Veli 8 Gy
γH2AX	1	1.09 ±0.3	0.58 ±0.3	3.45 ±0.1	3.78 ±1.4	2.55 ±1.6	4.27 ±0.3	3.17 ±0.4	4.88 ±0.7
p-ATM	1	0.77 ±0.1	0.89 ±0.1	2.54 ±0.6	3.87 ±1.6	3.51 ±1.1	3.82 ±2.7	5.31 ±2.1	2.1 ±0.3
p-BRCA	1	1.05 ±0.6	1.14 ±0.7	4.16 ±0.6	7.25 ±3.6	7.25 ±3.07	5.13 ±2.2	7.83 ±4.9	4.45 ±2.0
p-p53	1	1.58 ±0.3	1.88 ±0.8	3.57 ±1.6	10.61 ±0.2	9.08 ±1.4	6.40 ±0.7	6.3 ±0.4	5.85 ±2.7
Rad51	1	1.21 ±0.0	1.51 ±0.0	1.49 ±0.4	1.88 ±1.0	1.85 ±0.2	1.31 ±0.0	2.03 ±0.1	1.09 ±0.4

SW-1353 24h	ctrl 0 Gy	Ola 0 Gy	Veli 0 Gy	ctrl 4 Gy	Ola 4 Gy	Veli 4 Gy	ctrl 8 Gy	Ola 8 Gy	Veli 8 Gy
γH2AX	1	1.62 ±0.6	1.47 ±0.4	2.03 ±0.5	2.34 ±0.4	1.84 ±0.7	2.32 ±0.1	2.11 ±0.3	2.89 ±0.4
p-ATM	1	1.60 ±0.2	1.55 ±0.1	1.69 ±0.1	2.60 ±0.2	2.77 ±1.9	2.73 ±1.3	2.49 ±2.4	5.32 ±2.5
p-BRCA	1	2.98 ±0.3	5.19 ±0.2	1.69 ±0.2	4.21 ±1.9	3.04 ±0.9	3.26 ±1.6	1.25 ±0.3	3.48 ±1.0
p-p53	1	2.18 ±0.7	0.91 ±0.1	2.04 ±0.9	10.69 ±3.5	5.15 ±2.6	6.85 ±2.3	5.36 ±1.3	5.41 ±2.2
Rad51	1	0.96 ±0.1	0.95 ±0.2	0.89 ±0.1	1.23 ±0.1	1.05 ±0.2	1.24 ±0.1	1.29 ±0.3	1.21 ±0.1

Table S1. Protein phosphorylation pattern after combined treatment of 10 µM Olaparib or Veliparib treatment and 0 Gy (non-IR controls), 4 Gy, and 8 Gy proton IR of SW-1353 chondrosarcoma cells. Whole cell proteins were isolated 1 h and 24 h after IR. All changes were presented as fold change (Δ-ratio) normalised to non-IR controls (mean ± SD of n = 3). Representative blot are shown in Figure 2A.

SW-1353 1h	ctrl 0 Gy	Ku 0 Gy	VE 0 Gy	ctrl 4 Gy	Ku 4 Gy	VE 4 Gy	ctrl 8 Gy	Ku 8 Gy	VE 8 Gy
γH2AX	1	0.67 ±0.1	1.14 ±0.4	4.60 ±1.8	1.97 ±0.8	1.58 ±0.8	6.27 ±1.7	2.08 ±0.7	5.03 ±2.3
p-ATM	1	0.57 ±0.0	1.99 ±0.4	7.93 ±3.08	1.71 ±0.2	12.08 ±4.1	12.50 ±6.1	2.75 ±0.8	14.59 ±5.4
p-BRCA	1	1.59 ±0.1	1.64 ±0.6	3.05 ±0.1	5.88 ±2.64	7.11 ±1.86	6.88 ±2.35	5.33 ±2.79	6.61 ±2.19
p-p53	1	1.25 ±0.1	1.33 ±0.3	6.76 ±1.4	1.01 ±0.1	5.98 ±1.4	5.04 ±1.0	0.41 ±1.6	2.65 ±0.4
Rad51	1	1.03 ±0.3	1.41 ±0.1	1.69 ±0.5	0.78 ±0.1	1.25 ±0.0	1.06 ±0.1	0.97 ±0.1	0.67 ±0.1

SW-1353 24h	ctrl 0 Gy	Ku 0 Gy	VE 0 Gy	ctrl 4 Gy	Ku 4 Gy	VE 4 Gy	ctrl 8 Gy	Ku 8 Gy	VE 8 Gy
γH2AX	1	1.38 ±0.3	2.32 ±0.2	2.10 ±0.5	1.47 ±0.7	3.69 ±1.5	1.57 ±0.1	0.97 ±0.1	3.88 ±1.8
p-ATM	1	0.88 ±0.3	1.35 ±0.0	1.59 ±0.8	1.37 ±0.1	4.17 ±1.7	3.40 ±2.5	1.25 ±0.1	4.18 ±0.2
p-BRCA	1	2.65 ±0.26	1.21 ±0.33	1.66 ±0.15	3.09 ±1.14	1.48 ±0.48	1.39 ±0.31	1.25 ±0.69	1.00 ±0.17
p-p53	1	1.12 ±0.0	1.12 ±0.0	1.41 ±0.0	1.43 ±0.3	2.95 ±0.1	4.45 ±0.7	0.89 ±0.2	2.04 ±1.1
Rad51	1	1.19 ±0.3	0.79 ±0.2	1.51 ±0.3	1.53 ±0.9	1.14 ±0.4	1.99 ±0.2	0.89 ±0.0	0.83 ±0.0

Table S2. Protein phosphorylation pattern after combined treatment of 10 μM Ku-55933 and VE-821 treatment and 0 Gy (non-IR controls), 4 Gy, and 8 Gy proton IR of SW-1353 chondrosarcoma cells. Whole cell proteins were isolated 1 h and 24 h after IR. All changes were presented as fold change (Δ-ratio) normalised to non-IR controls (mean ± SD of n = 3). Representative blot are shown in Figure 2B.

Cal78 1h	ctrl 0 Gy	Ola 0 Gy	Veli 0 Gy	ctrl 4 Gy	Ola 4 Gy	Veli 4 Gy	ctrl 8 Gy	Ola 8 Gy	Veli 8 Gy
γH2AX	1	0.96 ±0.6	0.58 ±0.3	3.84 ±0.2	3.42 ±0.9	3.44 ±0.3	5.29 ±1.4	6.22 ±1.4	5.80 ±1.7
p-ATM	1	1.25 ±0.5	1.35 ±0.5	3.10 ±1.8	2.91 ±0.9	2.67 ±1.2	2.92 ±1.1	4.66 ±2.4	3.30 ±1.3
p-BRCA	1	2.10 ±0.6	2.13 ±0.6	2.59 ±0.1	5.00 ±1.2	3.74 ±0.3	2.77 ±0.8	4.28 ±1.3	3.24 ±0.7
p-p53	1	2.17 ±0.6	1.56 ±0.3	3.76 ±1.3	5.14 ±3.1	4.49 ±1.5	4.37 ±2.5	4.55 ±3.5	4.22 ±1.38
Rad51	1	1.46 ±0.6	1.64 ±0.7	1.66 ±0.5	2.48 ±1.5	2.97 ±1.3	1.16 ±0.5	1.58 ±1.0	1.83 ±0.4

Cal78 24h	ctrl 0 Gy	Ola 0 Gy	Veli 0 Gy	ctrl 4 Gy	Ola 4 Gy	Veli 4 Gy	ctrl 8 Gy	Ola 8 Gy	Veli 8 Gy
γH2AX	1	2.93 ±0.4	1.57 ±0.5	2.26 ±0.1	3.75 ±0.7	2.07 ±1.3	2.52 ±0.2	2.03 ±0.1	2.47 ±0.6
p-ATM	1	4.07 ±0.8	219 ±0.4	1.99 ±0.2	3.44 ±0.2	3.72 ±1.0	3.68 ±1.1	1.98 ±0.5	2.44 ±0.7
p-BRCA	1	3.42 ±1.4	2.66 ±0.6	1.54 ±0.3	4.23 ±0.4	3.59 ±1.60	2.73 ±1.0	0.94 ±0.1	3.03 ±0.5
p-p53	1	3.13 ±0.3	1.48 ±0.2	1.32 ±0.2	4.87 ±1.3	3.51 ±0.9	5.24 ±1.2	1.92 ±1.1	7.54 ±3.1
Rad51	1	1.77 ±0.5	1.37 ±0.3	1.76 ±0.3	1.77 ±0.1	2.93 ±1.7	2.37 ±0.6	2.35 ±0.6	2.38 ±0.5

Table S3. Protein phosphorylation pattern after combined treatment of 10 µM Olaparib or Veliparib treatment and 0 Gy (non-IR controls), 4 Gy, and 8 Gy proton IR of Cal78 chondrosarcoma cells. Whole cell proteins were isolated 1 h and 24 h after IR. All changes were presented as fold change (Δ-ratio) normalised to non-IR controls (mean ± SD of n = 3). Representative blot are shown in Figure 2C.

Cal78 1h	ctrl 0 Gy	Ku 0 Gy	VE 0 Gy	ctrl 4 Gy	Ku 4 Gy	VE 4 Gy	ctrl 8 Gy	Ku 8 Gy	VE 8 Gy
γH2AX	1	1.52 ±0.8	3.48 ±0.9	3.22 ±1.14	3.47 ±1.6	7.43 ±2.8	5.49 ±2.6	3.80 ±1.8	6.80 ±2.2
p-ATM	1	1.24 ±0.5	1.35 ±0.35	3.01 ±1.80	2.91 ±0.91	2.67 ±1.26	2.92 ±1.06	4.66 ±2.03	3.30 ±1.37
p-BRCA	1	1.66 ±0.6	1.67 ±0.7	2.67 ±0.6	4.45 ±1.79	2.72 ±1.3	2.94 ±1.4	3.50 ±1.9	2.31 ±0.9
p-p53	1	0.84 ±0.2	1.51 ±0.5	3.74 ±1.4	0.83 ±0.1	5.05 ±1.4	5.03 ±1.2	1.45 ±0.0	9.48 ±1.8
Rad51	1	0.96 ±0.3	0.76 ±0.3	2.02 ±0.1	1.55 ±0.2	1.25 ±0.3	2.93 ±0.1	1.93 ±1.2	0.89 ±0.1

Cal78 24h	ctrl 0 Gy	Ku 0 Gy	VE 0 Gy	ctrl 4 Gy	Ku 4 Gy	VE 4 Gy	ctrl 8 Gy	Ku 8 Gy	VE 8 Gy
γH2AX	1	0.94 ±0.1	3.57 ±0.6	2.82 ±0.7	1.47 ±0.6	4.81 ±1.1	1.56 ±0.3	1.60 ±0.1	6.03 ±3.4
p-ATM	1	4.07 ±0.78	2.19 ±0.38	1.99 ±0.23	3.44 ±0.18	3.72 ±1.05	3.68 ±1.12	0.98 ±0.53	1.44 ±0.66
p-BRCA	1	1.73 ±0.4	1.83 ±0.6	2.24 ±0.5	3.28 ±1.2	2.49 ±1.0	2.38 ±1.0	1.23 ±0.7	0.98 ±0.9
p-p53	1	0.67 ±0.4	0.72 ±0.3	1.32 ±0.2	0.89 ±0.2	1.39 ±0.8	3.71 ±1.2	2.82 ±1.6	2.41 ±1.1
Rad51	1	0.80 ±0.1	0.69 ±0.1	1.78 ±0.6	1.77 ±0.5	1.73 ±0.4	2.97 ±0.2	2.57 ±0.3	0.99 ±0.1

Table S4. Protein phosphorylation pattern after combined treatment of 10 µM Ku-55933 and VE-821 treatment and 0 Gy (non-IR controls), 4 Gy, and 8 Gy proton IR of Cal78 chondrosarcoma cells. Whole cell proteins were isolated 1 h and 24 h after IR. All changes were presented as fold change (Δ-ratio) normalised to non-IR controls (mean ± SD of *n* = 3). Representative blot are shown in Figure 2D.

	SW-1353 1 h	ctrl 0 Gy	VE-821 0 Gy	X-ray 4 Gy	proton 4 Gy	C-ions 4 Gy	VE-821 + X-ray 4 Gy	VE-821 + proton 4 Gy	VE-821 + C-ions 4 Gy
Fig. 5A	p-STAT3	1	1.0±0.1	0.1±0.0	0.1±0.0	0.1±0.0	0.1±0.0	0.1±0.0	0.1±0.0
	p-JNK	1	1.0±0.5	1.0±0.1	1.8±0.6	1.5±0.5	3.8±0.7	3.5±0.9	4.6±1.2
	p-p38	1	1.3±0.1	1.7±0.1	1.7±0.1	2.7±1.1	4.1±0.2	4.5±0.6	4.8±1.1
Fig. 6A	γH2AX	1	2.5±0.5	1.1±0.2	2.6±0.8	2.5±0.2	1.9±0.3	3.0±0.5	4.0±1.1
	Rad51	1	0.8±0.1	1.0±0.5	0.7±0.2	0.8±0.0	0.7±0.2	0.9±0.2	1.0±0.1
	MSH3	1	1.0±0.3	1.1±0.3	1.7±0.6	2.6±0.4	1.3±0.0	0.7±0.4	0.9±0.1
	XPC	1	0.9±0.4	2.2±0.4	2.1±0.2	2.3±0.3	1.9±0.2	1.3±0.7	1.2±0.1
	p-CHK1	1	0.6±0.1	1.3±0.3	2.6±0.8	3.1±0.1	0.4±0.1	0.6±0.2	0.7±0.2
	p-CHK2	1	1.0±0.0	5.1±0.9	5.2±0.3	6.3±1.3	5.7±0.9	5.1±0.4	6.2±1.1
	p-BRCA	1	1.6±0.8	3.3±1.2	3.9±1.7	4.1±0.1	1.5±0.1	2.3±0.2	2.3±0.4
	p-p53	1	1.0±0.3	5.2±0.2	5.8±1.0	5.7±1.1	4.3±0.1	4.1±0.4	4.8±0.6

	SW-1353 24 h	ctrl 0 Gy	VE-821 0 Gy	X-ray 4 Gy	proton 4 Gy	C-ions 4 Gy	VE-821 + X-ray 4 Gy	VE-821 + proton 4 Gy	VE-821 + C-ions 4 Gy
Fig. 5C	caspase 8	1	1.0±0.1	0.6±0.3	0.5±0.3	0.4±0.1	0.5±0.1	0.6±0.2	0.9±0.2
	caspase 9	1	1.1±0.1	0.8±0.2	0.7±04	0.7±0.3	1.0±0.1	1.0±0.0	1.0±0.0
	cleaved caspase 3	1	1.8±0.4	1.2±0.5	0.8±0.4	1.2±0.3	1.7±0.6	1.8±0.6	1.9±0.5
	survivin	1	0.8±0.1	0.8±0.1	1-3±0.2	1.4±0.2	0.5±0.0	0.4±0.2	0.3±0.0
Fig. 6A	γH2AX	1	2.8±0.3	1.0±0.0	1.0±0.1	1.2±0.4	3.5±0.2	3.7±0.2	4.8±0.6
	Rad51	1	1.0±0.3	0.6±0.1	0.7±0.0	1.1±0.3	0.7±0.1	0.7±0.1	0.4±0.1
	MSH3	1	0.7±0.1	0.9±0.0	0.9±0.0	1.1±0.4	0.6±0.1	0.6±0.2	0.4±0.0
	XPC	1	1.3±0.1	1.5±0.3	1.5±0.0	1.6±0.1	0.8±0.4	0.5±0.1	0.6±0.2
	p-CHK1	1	1.2±0.1	1.8±0.6	2.4±0.3	4.9±0.6	0.7±0.0	0.6±0.0	0.3±0.1
	p-CHK2	1	4.4±0.7	1.9±0.7	2.3±0.8	2.4±0.6	2.8±0.8	3.3±0.7	2.5±0.3
	p-BRCA	1	0.8±0.3	0.6±0.0	1.0±0.3	1.2±0.6	0.3±0.1	0.4±0.2	0.1±0.0
	p-p53	1	4.3±0.8	1.5±0.1	2.9±1.1	5.8±0.9	7.2±1.5	7.9±1.9	9.8±1.3

	SW-1353 72 h	ctrl 0 Gy	VE-821 0 Gy	X-ray 4 Gy	proton 4 Gy	C-ions 4 Gy	VE-821 + X-ray 4 Gy	VE-821 + proton 4 Gy	VE-821 + C-ions 4 Gy
Fig. 5C	caspase 8	1	1.2±0.1	0.7±0.1	0.6±0.1	0.6±0.0	0.6±0.0	0.6±0.1	0.8±0.1
	caspase 9	1	1.8±0.7	0.8±0.1	1.0±0.1	1.0±0.2	1.6±0.4	1.7±0.3	1.9±0.6
	cleaved caspase 3	1	0.9±0.2	1.3±0.5	1.3±0.0	1.2±0.1	1.3±0.2	1.6±0.6	1.7±0.6
	survivin	1	0.9±0.1	0.9±0.1	0.7±0.2	0.6±0.0	0.5±0.1	0.4±0.1	0.3±0.1
Fig. 6A	γH2AX	1	3.7±1.1	1.2±0.1	1.5±0.4	2.0±0.1	4.7±1.0	5.4±1.4	4.7±1.1
	Rad51	1	1.1±0.1	0.9±0.0	0.6±0.1	0.8±0.3	0.7±0.0	0.5±0.1	0.3±0.1
	MSH3	1	0.5±0.1	1.0±0.3	0.8±0.3	0.5±0.2	0.4±0.2	0.3±0.1	0.2±0.0
	XPC	1	1.2±0.5	1.6±0.1	1.4±0.3	1.3±0.5	1.5±0.4	0.8±0.3	0.4±0.2
	p-CHK1	1	1.1±0.1	0.8±0.2	0.7±0.1	1.2±0.3	0.8±0.2	0.8±0.2	0.5±0.2
	p-CHK2	1	2.5±0.0	0.3±0.1	0.5±0.1	0.8±0.1	3.7±1.1	4.1±1.4	7.3±1.8
	p-BRCA	1	1.3±0.2	1.0±0.2	1.0±0.4	0.6±0.1	0.8±0.0	0.8±0.1	0.3±0.1
	p-p53	1	2.6±0.9	0.9±0.1	1.5±0.3	1.9±0.3	4.1±1.2	5.9±0.7	6.3±0.7

Table S5. Protein expression and phosphorylation pattern after combined treatment of 10 µM VE-821 treatment and 0 Gy (non-IR controls) or 4 Gy X-ray/proton/C-ions IR of SW-1353 chondrosarcoma cells. Whole cell proteins were isolated 1 h, 24 h, and 72 h after IR. All changes were presented as fold change (Δ-ratio) normalised to non-IR controls (mean ± SD of n = 3). Representative blot are shown in Figure 5A, 5C and 6A.

	Cal78 1 h	ctrl 0 Gy	VE-821 0 Gy	X-ray 4 Gy	proton 4 Gy	C-ions 4 Gy	VE-821 + X-ray 4 Gy	VE-821 + proton 4 Gy	VE-821 + C-ions 4 Gy
Fig. 5B	p-STAT3	1	0.2±0.0	0.3±0.1	0.2±0.1	0.2±0.1	0.1±0.1	0.1±0.0	0.1±0.0
	p-JNK	1	1.5±0.2	1.7±0.5	1.5±0.9	3.8±1.0	3.7±0.8	2.9±0.9	4.5±1.1
	p-p38	1	2.2±0.5	1.4±0.4	1.5±0.8	1.3±0.1	4.4±1.5	5.9±1.6	5.4±1.9
Fig. 6B	γH2AX	1	3.3±1.5	2.1±0.7	3.3±1.2	4.1±0.8	4.6±1.6	3.2±1.1	4.4±0.7
	Rad51	1	0.7±0.2	1.3±0.3	1.3±0.6	2.4±1.0	0.7±0.5	0.6±0.4	0.5±0.2
	MSH3	1	0.6±0.2	1.0±0.2	0.9±0.3	0.7±0.0	0.5±0.1	0.5±0.1	0.5±0.2
	XPC	1	1.2±0.2	1.2±0.1	1.0±0.3	0.9±0.2	0.8±0.0	0.8±0.1	0.7±0.2
	p-CHK1	1	0.2±0.1	1.0±0.2	1.1±0.4	2.3±0.2	0.1±0.0	0.1±0.0	0.1±0.0
	p-CHK2	1	1.1±0.2	3.5±0.6	3.2±0.8	2.9±0.8	1.5±0.7	1.1±0.4	1.0±0.3
	p-BRCA	1	0.4±0.3	1.2±0.3	1.3±0.4	1.3±0.6	0.2±0.0	0.2±0.1	0.2±0.1
	p-p53	1	2.2±0.7	2.7±0.5	4.2±1.1	2.1±0.5	3.9±0.6	3.6±0.9	2.3±1.0

	Cal78 24 h	ctrl 0 Gy	VE-821 0 Gy	X-ray 4 Gy	proton 4 Gy	C-ions 4 Gy	VE-821 + X-ray 4 Gy	VE-821 + proton 4 Gy	VE-821 + C-ions 4 Gy
Fig. 5D	caspase 8	1	0.9±0.2	0.9±0.2	0.8±0.1	1.1±0.3	1.6±0.4	1.5±0.7	1.8±0.4
	caspase 9	1	0.8±0.3	1.1±0.3	0.7±0.3	0.8±0.3	0.6±0.2	0.9±0.2	0.9±0.3
	cleaved caspase 3	1	2.0±0.8	0.8±0.3	0.7±0.7	0.7±0.3	1.3±0.1	2.5±0.8	3.9±1.3
	survivin	1	1.1±0.1	1.8±0.5	1.7±0.4	1.1±0.5	0.8±0.3	0.7±0.2	0.4±0.1
Fig. 6B	γH2AX	1	3.0±0.9	0.8±0.1	0.8±0.1	1.0±0.3	3.7±1.8	3.0±1.3	4.1±1.4
	Rad51	1	0.7±0.2	1.3±0.6	1.5±0.7	1.7±0.2	0.9±0.3	0.8±0.5	1.1±0.7
	MSH3	1	0.8±0.3	1.1±0.1	0.9±0.1	1.5±0.2	1.0±0.4	0.8±0.5	0.8±0.1
	XPC	1	1.1±0.8	0.9±0.4	0.6±0.1	0.7±0.1	0.6±0.4	0.4±0.1	0.3±0.1
	p-CHK1	1	0.3±0.2	1.6±0.6	2.8±0.8	4.4±0.7	0.2±0.2	0.2±0.1	0.2±0.1
	p-CHK2	1	1.1±0.7	1.3±0.8	1.7±0.6	2.0±0.8	0.5±0.2	0.2±0.0	0.2±0.1
	p-BRCA	1	1.8±0.8	0.9±0.1	1.1±0.3	1.2±0.7	0.9±0.3	1.6±0.6	0.9±0.1
	p-p53	1	2.3±0.6	1.8±0.8	2.8±0.3	7.2±0.1	6.7±1.8	6.4±0.2	6.4±1.2

	Cal78 72 h	ctrl 0 Gy	VE-821 0 Gy	X-ray 4 Gy	proton 4 Gy	C-ions 4 Gy	VE-821 + X-ray 4 Gy	VE-821 + proton 4 Gy	VE-821 + C-ions 4 Gy
Fig. 5D	caspase 8	1	2.1±0.7	0.6±0.2	1.2±0.4	1.5±0.4	2.6±0.1	2.8±0.1	2.6±0.2
	caspase 9	1	0.9±0.2	1.4±0.0	1.2±0.1	1.3±0.3	0.8±0.3	0.8±0.1	0.7±0.4
	cleaved caspase 3	1	3.2±1.1	1.0±0.0	1.2±0.1	0.9±0.3	3.5±1.2	3.5±1.0	3.8±0.9
	survivin	1	0.6±0.2	2.5±0.5	2.1±0.5	1.9±0.2	1.0±0.1	1.1±0.3	1.2±0.2
Fig. 6B	γH2AX	1	4.2±0.3	1.4±0.1	1.8±0.2	1.9±1.1	5.4±0.3	4.5±1.2	4.4±0.6
	Rad51	1	0.8±0.4	1.8±0.7	1.4±0.3	1.5±0.2	0.6±0.1	0.6±0.4	0.4±0.1
	MSH3	1	0.7±0.2	2.5±0.4	2.2±0.6	2.5±0.7	0.5±0.0	0.4±0.1	0.4±0.1
	XPC	1	0.9±0.2	2.4±0.3	1.7±0.1	2.4±0.6	0.8±0.0	0.8±0.1	0.8±0.1
	p-CHK1	1	1.0±0.1	1.4±0.4	2.4±0.6	4.6±1.3	0.8±0.2	0.9±0.0	0.9±0.2
	p-CHK2	1	0.7±0.4	1.2±0.1	1.1±0.2	1.7±0.2	0.6±0.3	0.4±0.2	0.3±0.1
	p-BRCA	1	1.6±0.5	1.3±0.2	1.5±0.6	1.6±0.3	0.7±0.2	0.6±0.1	0.4±0.1
	p-p53	1	2.5±0.3	1.0±0.1	0.8±0.2	0.6±0.4	1.8±0.5	1.7±0.3	2.4±0.8

Table S6. Protein expression and phosphorylation pattern after combined treatment of 10 µM VE-821 treatment and 0 Gy (non-IR controls) or 4 Gy X-ray/proton/C-ions IR of Cal78 chondrosarcoma cells. Whole cell proteins were isolated 1 h, 24 h, and 72 h after IR. All changes were presented as fold change (Δ-ratio) normalised to non-IR controls (mean ± SD of $n = 3$). Representative blot are shown in Figure 5B, 5D and 6B.
